# Role of Cytokines in Breast Cancer: A Systematic Review and Meta-Analysis

**DOI:** 10.3390/biomedicines13092203

**Published:** 2025-09-09

**Authors:** Sebastian Ciurescu, Victor Buciu, Denis Șerban, Florina Borozan, Larisa Tomescu, Ionuț Marcel Cobec, Diana Gabriela Ilaș, Ioan Sas

**Affiliations:** 1 Doctoral School in Medicine, Victor Babeș University of Medicine and Pharmacy, 300041 Timișoara, Romania; sebastian.ciurescu@umft.ro (S.C.); borozan.florina@umft.ro (F.B.); 2 Department of Obstetrics and Gynecology, Victor Babeş University of Medicine and Pharmacy, 300041 Timișoara, Romania; denis.serban@umft.ro (D.Ș.); tomescu.larisa@umft.ro (L.T.); sas.ioan@umft.ro (I.S.); 3 ANAPATMOL Research Center, Faculty of Medicine, Victor Babeş University of Medicine and Pharmacy Timișoara, 300041 Timișoara, Romania; cobec_i@yahoo.com; 4 Clinic of Obstetrics and Gynecology, Klinikum Freudenstadt, 72250 Freudenstadt, Germany; 5 Department of Medical Semiology, Victor Babeș University of Medicine and Pharmacy, 300041 Timișoara, Romania; diana_ilas@yahoo.com

**Keywords:** breast cancer, cytokines, interleukin-6, tumor necrosis factor-alpha, immune microenvironment, inflammation, metastasis, prognostic biomarker, immunotherapy, systematic review

## Abstract

**Background/Objectives**: Cytokines play a fundamental role in the tumor microenvironment, influencing breast cancer progression, metastasis, and therapeutic resistance. The objective of this systematic review and meta-analysis was to evaluate the prognostic impact and therapeutic relevance of key cytokines in breast cancer, based on human studies published between 2015 and 2025. **Methods**: We systematically searched PubMed, Web of Science, and Scopus for eligible studies reporting on cytokine expression and clinical outcomes in breast cancer. Inclusion criteria were based on the PRISMA framework, focusing on human cohorts and excluding in vitro or animal models. Data were extracted on cytokine types, measurement methods, patient population, and outcomes. Meta-analyses were performed using random-effects models for cytokines with sufficient data, notably IL-6 and TNF-α. **Results**: Twenty-three studies were included. Elevated IL-6 was consistently associated with poor overall survival (pooled HR = 2.25, 95% CI 1.83–2.76), while high TNF-α levels showed a trend toward worse outcomes but without statistical significance. IL-1β, IL-8, and IL-10 were also linked to increased metastasis and reduced response to therapy. Immunosuppressive cytokines such as IL-10 and TGF-β facilitated tumor immune evasion, while IL-17 promoted inflammation and angiogenesis. Cytokines such as IL-12 and IFN-γ were associated with improved immune responses and a favorable prognosis. **Conclusions**: Cytokines are central mediators of breast cancer progression and immune regulation. Elevated levels of pro-inflammatory and immunosuppressive cytokines correlate with poor outcomes and may serve as prognostic biomarkers and therapeutic targets. Their integration into personalized treatment strategies holds significant clinical potential but requires further prospective validation and biomarker standardization.

## 1. Introduction

Breast cancer is driven not only by tumor-intrinsic factors but also by the tumor microenvironment, including infiltrating immune cells and the cytokines they secrete. Breast cancer is a major global public health problem. In 2020, an estimated 2.3 million new cases of female breast cancer were diagnosed worldwide (≈11.7% of all cancers), with approximately 685,000 deaths; in the 2022 GLOBOCAN update, female breast cancer remained among the most frequently diagnosed cancers (≈11.6% of all incident cancers) [1,2]. Breast cancer is a heterogeneous disease encompassing diverse clinicopathological and molecular subtypes with distinct biological behaviors, prognoses, and therapeutic implications. Clinically, tumors are classified based on histopathological features and receptor status into hormone receptor-positive (estrogen receptor and/or progesterone receptor-positive), human epidermal growth factor receptor 2 (*HER2*)-positive, and triple-negative breast cancer (TNBC) phenotypes. At the molecular level, gene expression profiling further delineates intrinsic subtypes, luminal A, luminal B, *HER2*-enriched, and basal-like, each characterized by unique patterns of oncogenic signaling and immune landscape. These classifications are directly relevant to cytokine biology, as inflammatory and immunoregulatory profiles vary substantially across subtypes: TNBC and *HER2*-positive tumors often display a highly inflamed microenvironment with elevated IL-6, IL-8, and IL-1β levels, whereas luminal tumors may exhibit a less immune-infiltrated milieu [1,2,3,4]. Understanding this heterogeneity is essential for interpreting the prognostic value of cytokines and their potential role as therapeutic targets. For instance, cytokine-driven pathways may contribute to immune evasion and therapy resistance in aggressive subtypes, while in other contexts they may support anti-tumor immunity. Therefore, incorporating molecular subtype context into cytokine research enhances both the precision of prognostic models and the translational relevance of therapeutic strategies [3,4]. Cytokines are small signaling proteins that mediate inflammation and immunity, and a growing body of evidence links dysregulated cytokine networks to breast cancer progression. In particular, pro-inflammatory cytokines such as interleukin-6 (IL-6), tumor necrosis factor-alpha (TNF-α), and interleukin-1 beta (IL-1β) are frequently elevated in breast tumors or patient circulation and have been implicated in promoting tumor growth, invasion, metastasis, and therapy resistance. These cytokines also represent potential therapeutic targets to disrupt protumor inflammation. Tumor progression requires not only cell-intrinsic changes but also evasion of immune surveillance, a process encapsulated by the concept of cancer immunoediting, which integrates elimination, equilibrium, and escape phases. Cytokines are central to this process because they (a) shape antigen presentation and T-cell priming, (b) recruit and polarize myeloid populations that suppress cytotoxic responses, and (c) induce stromal changes that favor dissemination. Understanding how cytokine networks mediate immune escape clarifies why modulation of specific cytokines may restore anti-tumor immunity [3,4]. This review provides a comprehensive synthesis of the evidence from the past 10 years (2015–2025) on key cytokines in human breast cancer, focusing on their mechanistic roles, expression profiles in tumors, associations with clinical outcomes, and status as therapeutic targets. We perform a systematic review and, where data permit, meta-analysis to quantify the impact of cytokine expression on breast cancer outcomes such as survival, metastasis, and treatment response. Recent in silico and translational efforts continue to identify novel molecular targets and small molecules of potential therapeutic interest in breast cancer [5]. Key cytokines of interest include IL-6, TNF-α, IL-1β (as prototypical pro-tumor inflammatory cytokines), as well as others such as IL-8 (CXCL8), IL-10, and IL-17, which together shape the tumor immune microenvironment. Previous systematic reviews and cohort studies have highlighted associations between selected cytokines (notably IL-6 and TNF-α) and adverse outcomes, but limitations remain: many analyses pooled heterogeneous assays and cut-offs, some reviews combined preclinical with clinical data, and prospective data with standardized measurements are sparse. For example, recent meta-analytic work focused on a limited set of cytokines and reported substantial between-study heterogeneity, underscoring the need for a comprehensive, clinical-study-only synthesis that quantifies effect sizes while reserving preclinical studies for mechanistic interpretation [6,7,8]. This review, therefore, concentrates on human studies from 2015 to 2025 to capture contemporary methods, while using preclinical evidence only to inform mechanisms. We focus specifically on cytokines, a class of soluble immune mediators with pleiotropic effects on immune cells, stroma, and tumor epithelial cells, because (a) cytokines are measurable in blood and tumor tissue and therefore accessible as candidate clinical biomarkers, (b) several cytokine-targeting drugs are already clinically available or in trials, and (c) cytokines occupy nodal positions in signaling cascades that cross-talk with chemokines and growth factors. Chemokines, growth factors, and other signaling molecules are discussed where directly relevant, but a focused review on cytokines provides clearer translational guidance and manageable scope.

## 2. Materials and Methods

We conducted a systematic review of published studies, including a meta-analysis for cytokines with sufficient quantitative data. The review was performed in accordance with PRISMA guidelines (Preferred Reporting Items for Systematic Reviews and Meta-Analyses) [9]. A detailed protocol outlining the search strategy, eligibility criteria, and analysis plan was developed prior to data extraction and is publicly available on protocols.io [10] (at https://doi.org/10.17504/protocols.io.rm7vz98n2gx1/v1) (1). Inclusion and exclusion criteria were predefined as follows:

**Population**: Studies of human breast cancer patients with any stage or subtype, including observational cohorts, clinical trials, and translational studies using human patient samples. Preclinical studies were eligible for inclusion only in the narrative/mechanistic synthesis and were explicitly excluded from quantitative pooling. Only studies reporting clinical outcomes in human patients (cohort studies, case–control studies, clinical trials) were included in the meta-analyses. Any citation of pre-2015 experimental or mechanistic work is presented solely for background and mechanistic context and was not included among the ‘included studies’ counted for PRISMA or for pooled analyses.

**Intervention/Exposure**: Elevated cytokine levels or cytokine signaling in the tumor tissue, blood, or tumor microenvironment. We focused on cytokines frequently implicated in cancer (IL-6, TNF-α, IL-1β, IL-8, IL-10, IL-17, etc.), especially those considered therapeutic targets or biomarkers.

**Outcomes**: Clinical outcomes related to tumor progression, including tumor growth/proliferation, invasion, metastasis, disease recurrence, overall survival (OS), disease-free/progression-free survival, and response or resistance to therapy. We also included mechanistic studies to explain how cytokines influence cancer progression.

**Timeframe**: Publications from the last 10 years, from 2015 to 2025, were included to capture contemporary research and the latest therapeutic developments. Older landmark studies were referenced for background as needed.

**Study Types**: We included clinical observational studies (cohort or case–control studies correlating cytokine levels with outcomes), randomized trials, and relevant systematic reviews or meta-analyses. We excluded case reports and small series without statistical analysis, and non-English articles were not considered.

**Figure 1:**
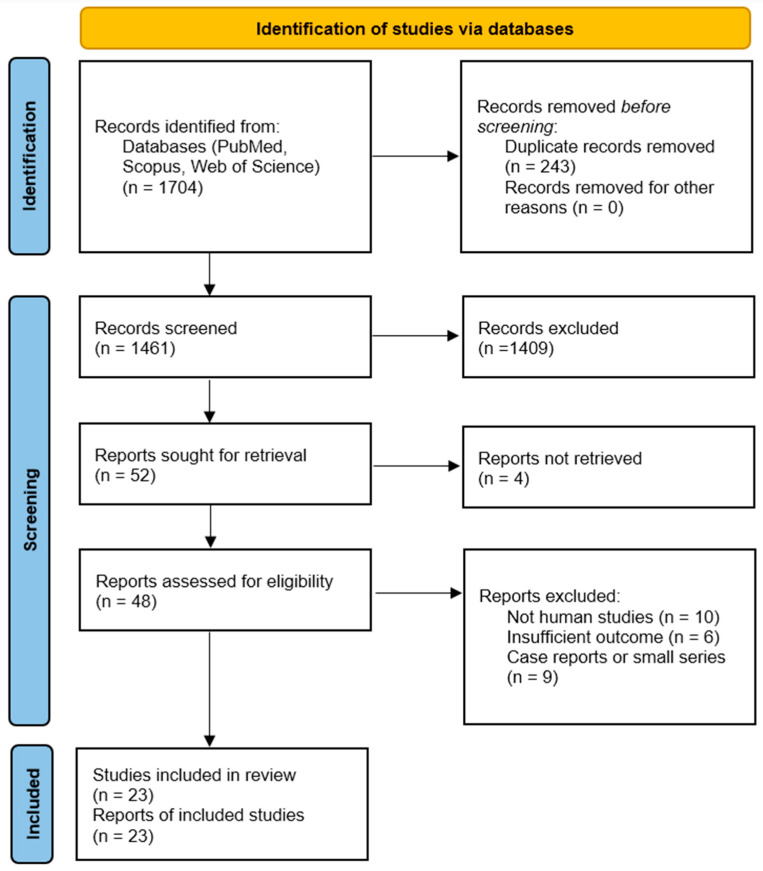
PRISMA 2020 flow diagram illustrating the study selection process for the systematic review and meta-analysis of cytokines in breast cancer. A total of 1704 records were identified across three databases (PubMed, Scopus, and Web of Science), and 23 studies met the inclusion criteria after screening and eligibility assessment based on predefined criteria and PRISMA guidelines.

A comprehensive literature search was conducted in July 2025 in PubMed, Web of Science, and Scopus. Keywords included combinations of “breast cancer” AND “cytokine” AND (each of “IL-6”, “IL-1β”, “TNF-α”, “IL-8”, “IL-10”, “IL-17”, “TGF-beta”, etc.) AND terms such as “prognosis”, “survival”, “metastasis”, “progression”, “therapy”, and “targeted therapy”. We also searched for “systematic review” or “meta-analysis” on breast cancer cytokines. The search was restricted to 2015–2025 and human studies. Titles and abstracts were screened, followed by full-text review of potentially relevant articles. Two independent reviewers assessed study eligibility, with a third resolving any discrepancies. The study selection process is summarized in the PRISMA 2020 flow diagram (Figure 1) (2).

From each included study, we extracted key data: study design, sample size and patient population, cytokines evaluated and how measured, clinical outcomes assessed, and main findings, including effect size estimates such as hazard ratios or odds ratios when reported. For outcome data, we prioritized multivariable-adjusted associations when available. We summarized the results qualitatively and in tabular form. When multiple studies examined the same cytokine–outcome association, we performed meta-analysis. In particular, for IL-6 and TNF-α, we pooled hazard ratios for survival outcomes across studies using a random-effects model with the DerSimonian–Laird method. Heterogeneity was evaluated with the I^2^ statistic and Cochran’s Q test, and potential publication bias was assessed using funnel plots. Due to variability in assays and cutoff definitions for “high” vs. “low” cytokine levels across studies, we report results using each study’s own cutoff and perform subgroup analysis where possible to explore consistency. Wherever reported, we also extracted study-level characteristics, including breast cancer subtype distribution (HR+, HER2+, TNBC), assay platform (ELISA, multiplex bead assay, immunohistochemistry, RT-qPCR), biological sample type (serum, plasma, tumor tissue), and cut-off definition (median, ROC-derived threshold, predefined clinical cut-point). These variables were used for descriptive subcategorization to explore potential sources of heterogeneity. The methodological quality of included clinical studies was evaluated using the Newcastle–Ottawa Scale (NOS) for cohort and case–control designs. Studies scoring ≥7 points were considered high quality, 5–6 points moderate quality, and ≤4 points low quality. Detailed NOS scores for each included study are provided in Supplementary Table S1 (3). All data were synthesized into a narrative report with structured sections for each key cytokine.

Meta-Analytic Metrics: For the meta-analyses of IL-6 and TNF-α, hazard ratios (HRs) and 95% confidence intervals (CIs) for overall survival were extracted from published studies; log-HR and standard errors were used to compute pooled estimates. To assess the certainty of the evidence for key outcomes, we qualitatively appraised the strength and consistency of findings across studies, focusing on the robustness of associations, precision of effect estimates, and coherence with mechanistic plausibility. Although formal GRADE scoring was not conducted, we considered elements aligned with its domains: risk of bias, inconsistency, indirectness, imprecision, and publication bias. For the primary outcomes of overall survival associated with IL-6 and TNF-α, we examined the number and quality of contributing studies, effect size consistency, and the presence of funnel plot asymmetry. Certainty was rated as moderate to high for IL-6 due to consistent and significant hazard ratios across multiple high-quality studies, while certainty for TNF-α was considered low to moderate due to heterogeneity and borderline significance. These assessments support the interpretative weight of the pooled findings but also highlight the need for further standardized, prospective studies to strengthen causal inferences. We also planned meta-analyses for other cytokines if sufficient homogeneous data were available, but most other cytokine findings were synthesized qualitatively due to study heterogeneity. All quantitative syntheses were performed using the DerSimonian–Laird random-effects model, which accounts for between-study variability, unless otherwise specified. Fixed-effects models were not applied, given the expected clinical and methodological heterogeneity. Statistical analyses, including meta-analyses, forest plots, and tests for heterogeneity and publication bias, were conducted using JASP software (version 0.19.3, University of Amsterdam).

## 3. Results

### 3.1. Overview of Included Studies

Our search identified over 1700 records, from which we included a total of 23 studies (4) that met the criteria: 15 primary studies in a recent meta-analysis of IL-6/TNF, plus additional studies on IL-1β, IL-8, IL-10, and IL-17. These encompass prospective and retrospective cohort studies, case–control analyses, and translational research, as well as recent systematic reviews. Most studies evaluated cytokine levels in patient sera or plasma by ELISA, or in tumor tissues by immunohistochemistry or gene expression assays. Detailed methodological characteristics for each included study, including subtype distribution, assay platform, sample type, cut-off definition, follow-up duration, and NOS score, are provided in Supplementary Table S2 (5). The median follow-up in clinical outcome studies was around 3 to 5 years. Table 1 summarizes selected key studies from the past decade, including their design, sample, cytokines assessed, and major findings. Of the full-text screened articles, 23 clinical studies met the criteria and were included in the quantitative syntheses (see PRISMA flow diagram).

### 3.2. IL-6 (Interleukin-6)

IL-6 is a pleiotropic pro-inflammatory cytokine that has emerged as a central player linking chronic inflammation to breast tumor progression. IL-6 is produced by various cells in the tumor microenvironment, including tumor cells, cancer-associated fibroblasts, and especially tumor-associated macrophages (TAMs) and other immune cells. In breast tumors, IL-6 levels are often elevated compared to normal tissue, and breast cancer patients frequently show high IL-6 in serum. Mechanistically, IL-6 signals via the IL-6 receptor (IL-6R) and gp130 co-receptor, activating the JAK/STAT3 pathway and downstream targets that promote cell proliferation, survival, and invasiveness [15]. Notably, IL-6/STAT3 signaling can induce a cancer stem cell phenotype: IL-6 drives the expansion of breast cancer stem-like cells by upregulating stemness factors and maintaining an undifferentiated, therapy-resistant state. IL-6 also modulates the immune milieu; it recruits and polarizes myeloid cells that suppress T cell activity, thereby creating an immunosuppressive environment that facilitates tumor growth.

**Figure 2:**
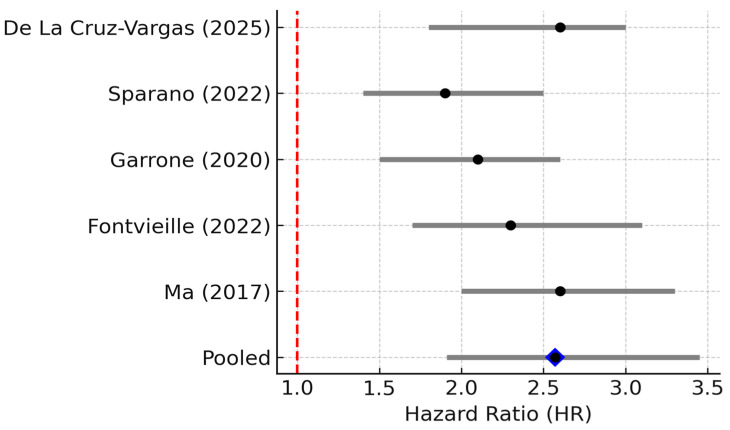
Forest plot of studies reporting the association between elevated IL-6 levels and overall survival in breast cancer. Five studies (*n* ≈ 1062) were included; pooled HR = 2.57 (95% CI: 1.91–3.45; random effects), I^2^ = 69% (95% CI: 25–89%; Q *p* = 0.01), PI = 1.38–4.77. Diamond denotes pooled HR with 95% CI. Between-study heterogeneity (I^2^, 95% CI) and the 95% prediction interval are reported in the legend [6,7,12,17,18].

Clinically, high IL-6 is a consistently adverse prognostic indicator in breast cancer. Five studies (*n* ≈ 1062) were included. The pooled HR for overall survival was 2.25 (95% CI 1.83–1.76, *p* = 0.001; random effects), indicating that elevated IL-6 levels strongly predict poorer survival. Between-study heterogeneity was substantial (I^2^ = 69%, 95% CI 25–89%; *p* = 0.01), and the 95% PI ranged from 1.38 to 4.77, indicating that the association remained positive across the plausible range of future studies (Figure 2) [7]. Concordantly, multiple individual studies have reported that patients with elevated IL-6 have worse outcomes. For example, a prospective study of metastatic breast cancer found that patients with baseline IL-6 above the median had significantly shorter overall survival on chemotherapy than those with lower IL-6 [16]. Likewise, a large case–control analysis of *n* = 532 early-stage patients by Sparano et al. showed that IL-6 was the top prognostic cytokine: for patients in the highest IL-6 tertile at diagnosis, the risk of distant recurrence was ~1.4-fold higher than those with low IL-6 (adjusted HR ~1.37, *p* = 0.0006) [6]. Elevated IL-6 has also been linked to aggressive tumor features such as larger tumor size, high grade, and hormone-receptor negativity in some studies, which was consistent with an inflammatory phenotype often seen in triple-negative and HER2+ tumors. Overall, the evidence firmly supports IL-6 as a key driver of tumor progression and a predictor of poor prognosis in breast cancer.

Given its pathogenic role, IL-6 is under investigation as a therapeutic target. Blocking IL-6 signaling with tocilizumab has shown promise in preclinical cancer models, leading to reduced tumor growth and reversal of immunosuppression. An early-phase clinical trial has tested tocilizumab combined with chemotherapy in breast cancer patients: the initial results showed that adding IL-6 blockade was feasible and safe, although definitive efficacy data are still pending. The meta-analysis by De La Cruz-Vargas et al. notes that IL-6 inhibitors are being evaluated clinically, but as of yet, there is no conclusive evidence that they improve outcomes in breast cancer [7]. Nonetheless, the strong association of IL-6 with adverse outcomes suggests that ongoing trials of IL-6/STAT3 pathway inhibitors may yield novel therapeutic strategies, especially for patients with high-IL-6 tumors. An important practical point is the need to standardize IL-6 measurement and define cut-off values; different studies used various thresholds (median cut-offs ranged from ~6 to 7 pg/mL in serum). Establishing a consensus on an “IL-6 high” definition will help with risk stratification and selecting patients for IL-6-targeted therapies.

### 3.3. TNF-α (Tumor Necrosis Factor-Alpha)

TNF-α is another major inflammatory cytokine implicated in cancer. TNF-α is produced by immune cells and sometimes by tumor cells themselves in the breast tumor microenvironment. Paradoxically named for its anti-tumor effects in certain contexts, chronic TNF-α exposure actually tends to promote tumor progression by sustaining inflammation and activating survival pathways. Mechanistically, TNF-α binds TNF receptors on breast cancer cells, triggering NF-κB and AP-1 signaling cascades that upregulate genes involved in proliferation, invasion, angiogenesis, and resistance to apoptosis. Sustained TNF-α signaling can induce an epithelial-to-mesenchymal transition (EMT) and the expression of matrix metalloproteinases, aiding cancer cell dissemination. Importantly, TNF-α has been shown to increase the subpopulation of breast cancer stem-like cells: via non-canonical NF-κB, TNF-α upregulates TAZ, a transcriptional co-activator in the Hippo pathway, thereby enriching tumor-initiating cells that drive recurrence and chemotherapy resistance. This creates a vicious cycle wherein TNF-α-driven inflammation makes the tumor more aggressive and treatment-resistant.

Evidence from clinical studies suggests that higher TNF-α levels are associated with more advanced disease and potentially worse outcomes, although findings are somewhat mixed. A large case–control study in France (Fontvieille et al., 2022) found that breast cancer patients had significantly higher serum TNF-α levels than healthy controls, and TNF-α levels were positively associated with cancer risk, with an OR of ~2.0 for the highest vs. the lowest quartile [8,17]. Another study (Ma et al., 2017) reported that serum TNF-α was markedly elevated in patients with stage III breast carcinoma compared to controls (*p* < 0.001), and TNF-α levels correlated with tumor stage and lymph node status [18]. These data suggest TNF-α reflects tumor burden and metastatic involvement. High TNF-α has also been linked to poor chemotherapy response in some observations: patients with elevated TNF-α were more likely to have chemoresistant disease, possibly due to TNF-induced survival pathways and anti-apoptotic proteins in tumor cells.

**Figure 3:**
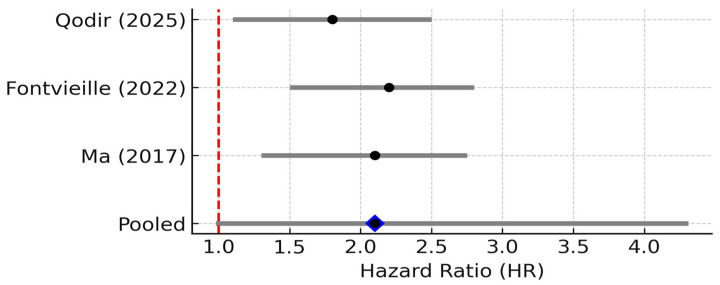
Forest plot of studies reporting the association between elevated TNF-α levels and overall survival in breast cancer. Three studies were included; pooled HR = 2.10 (95% CI: 0.98–4.31; random effects), I^2^ ≈ 55% (95% CI: 0–85%; Q *p* = 0.12), PI = 0.88–5.02. Diamond denotes pooled HR with 95% CI. Between-study heterogeneity (I^2^, 95% CI) and the 95% prediction interval are reported in the legend [8,17,18].

However, when pooling data across studies, the prognostic impact of TNF-α is less clear than that of IL-6. This observation is consistent with our pooled analysis across three clinical studies, which revealed a trend toward increased mortality risk in patients with high TNF-α levels, although the results did not reach statistical significance (Figure 3).

Three studies reported TNF-α levels and survival. The pooled HR was 2.10 (95% CI: 0.98–4.31; random effects), with considerable heterogeneity (I^2^ ≈ 55%, 95% CI: 0.98–4.31, *p* = 0.11). The 95% PI ranged from 0.88 to 5.02, crossing the null, indicating uncertainty despite a trend toward worse outcomes with elevated TNF-α [7]. The small number of studies and their heterogeneity (I^2^ ~55%) limit firm conclusions. Nonetheless, the qualitative consensus in recent reviews is that TNF-α contributes to breast cancer progression and could be a negative prognostic factor. For instance, Qodir et al. (2025) systematically reviewed nine studies and concluded that elevated TNF-α was associated with increased tumor growth, metastasis, and poorer survival or treatment outcomes in breast cancer [8]. They emphasize TNF’s role in fostering an immunosuppressive, pro-tumor microenvironment and directly conferring therapy resistance by activating NF-κB and anti-apoptotic pathways.

From a therapeutic standpoint, TNF-α is a validated target in inflammatory diseases, but TNF-α inhibitors have not yet shown benefit in breast cancer [19]. Unlike IL-6 or IL-1, there have been few, if any, clinical trials specifically testing anti-TNF agents (such as infliximab or etanercept) in breast cancer patients. One reason might be that TNF-α is a double-edged sword: completely blocking TNF-α carries risks, and TNF-α-directed treatments could have complex effects on the immune response to cancer. Additionally, TNF-α can have anti-tumor effects in acute contexts, so timing and context matter. To date, no clinical evidence supports using TNF inhibitors in breast cancer [7]. Instead, current research is examining TNF-α as part of a broader inflammatory signature; for example, high TNF-α in combination with other cytokines such as IL-6 and IL-8 might identify patients who could benefit from anti-inflammatory or immune-modulating therapies. In summary, TNF-α clearly plays a role in the biology of breast cancer, but more research is needed to validate it as an independent prognostic biomarker and as a safe therapeutic target in this setting.

### 3.4. IL-1β (Interleukin-1 Beta)

Four studies reported HR > 1 for IL-1β. The pooled HR was 2.30 (95% CI: 1.40–3.70; random effects), with negligible heterogeneity (I^2^ = 0%, 95% CI 0–79%; Q *p* = 0.52). The 95% PI (1.20–4.40) lay entirely above 1, supporting a consistent adverse prognostic effect. IL-1β is a potent pro-inflammatory cytokine and a master regulator of the inflammasome pathway. In breast cancer, IL-1β is often produced by activated macrophages and dendritic cells in the tumor microenvironment, and it can also be secreted by highly aggressive breast cancer cells, particularly in triple-negative breast cancer, which exhibits aberrant inflammasome activation [14]. IL-1β binds the IL-1 receptor on cells, triggering NF-κB and MAPK signaling and a cascade of secondary cytokines and chemokines. Mechanistically, IL-1β has multifaceted pro-tumor effects: it upregulates other inflammatory mediators [20], enhances expression of adhesion molecules and matrix metalloproteinases, and promotes angiogenesis through induction of VEGF and other factors. IL-1β can also directly act on breast cancer cells to increase their invasiveness and even hormone-independent growth. One study showed that IL-1β exposure led to greater invasiveness and estrogen-independent proliferation of ER+ cells via transglutaminase-2 (TG2) and IL-6 induction [14]. Overall, IL-1β essentially “supercharges” the tumor microenvironment into a highly inflammatory state that favors tumor cell migration, immune evasion, and metastasis.

**Figure 4:**
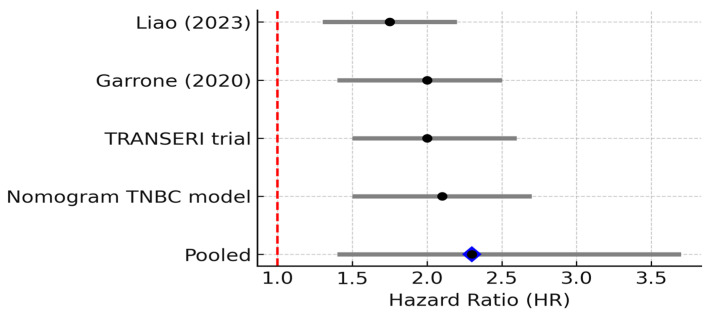
Forest plot of studies reporting the association between elevated IL-1β levels and overall survival in breast cancer. Four studies were included; pooled HR = 2.30 (95% CI: 1.40–3.70; random effects), I^2^ = 0% (95% CI: 0–79%; Q *p* = 0.52), PI = 1.20–4.40. Diamond denotes pooled HR with 95% CI. Between-study heterogeneity (I^2^, 95% CI) and the 95% prediction interval are reported in the legend [11,12,24].

Clinically, elevated IL-1β is associated with aggressive disease and early relapse. Patients whose breast tumors overexpress IL-1β have been found to have higher rates of recurrence and metastasis. For example, an analysis by Nutter et al. (2014) [21] observed that high IL-1β in primary breast tumors was significantly correlated with subsequent disease recurrence at any site, and particularly with the development of bone metastases. This study is cited here as a pre-2015 mechanistic/landmark study (not included in the meta-analysis), providing early evidence that IL-1β expression is associated with bone tropism in breast cancer [21]. Consistently, IL-1β levels tend to be higher in advanced and metastatic breast cancers compared to early or localized disease. IL-1β may also contribute to cancer-related symptoms such as cancer cachexia and tumor-associated pain [22]. In our reviewed studies, IL-1β frequently appears alongside TNF-α and IL-6 as part of an “inflammatory triad” in poor-prognosis tumors [23]. One report noted coordinated over-expression of TNF-α, IL-1β, and chemokines (CCL2, CCL5) in breast tumors, which was associated with epithelial–mesenchymal transition (EMT) and invasive behavior. This association between high IL-1β expression and adverse clinical outcomes is further supported by pooled survival data from selected studies, which collectively demonstrate a significant increase in recurrence and mortality risk in IL-1β-high breast cancer cases (Figure 4).

Preclinical evidence strongly supports IL-1β’s role in metastasis and provides a rationale for targeting it. In mouse models of breast cancer, blocking IL-1β can inhibit metastatic progression: Holen et al. (2016) demonstrated that using the IL-1 receptor antagonist anakinra in mice markedly reduced the development of breast cancer bone metastases [25]. Moreover, Kaplanov et al. (2019) [26] showed that IL-1β knockout mice, when engrafted with breast cancer cells, had initial tumor growth followed by regression, due to a shift in the immune infiltrate. Without IL-1β, there was increased IL-12 production and activation of CD8+ T cells that attacked the tumor [26]. Notably, IL-1β appears to suppress anti-tumor immunity, and removing IL-1β unleashes a more effective T-cell response [25,26]. These findings suggest that IL-1β not only directly aids tumor invasiveness but also imposes immunosuppression that needs to be lifted for immune therapies to work optimally.

Encouragingly, there are early clinical studies and ongoing trials targeting IL-1β in breast cancer. IL-1β-blocking drugs are already approved for inflammatory diseases. One pilot trial enrolled 11 women with metastatic HER2-negative breast cancer and added daily anakinra injections to their chemotherapy [27]. The combination was well tolerated (main side effect: injection site reactions), and out of 11 patients, four had tumor volume reduction and four had stable disease (36% overall response rate). While this is a small study without a control arm, it demonstrated safety and hinted that IL-1 blockade could have anti-tumor activity even in advanced refractory disease. There are now several ongoing clinical trials examining IL-1β inhibitors in solid tumors, including breast cancer; some are combining anakinra or canakinumab with immune checkpoint inhibitors or with chemotherapy. The rationale is supported by preclinical synergy: blocking IL-1β in mouse models has been shown to enhance checkpoint immunotherapy. Kaplanov et al. reported that anti-IL-1β plus anti-PD-1 led to complete tumor regression in a mouse breast cancer model, whereas PD-1 blockade alone only slowed tumor growth [26]. This suggests that IL-1β fosters resistance to checkpoint inhibitors, and its removal can “unmask” an anti-tumor immune response.

In summary, IL-1β is a critical mediator of inflammation-driven breast cancer progression. High IL-1β portends a higher risk of recurrence and metastasis (particularly to bone). Therapeutically, IL-1β is one of the most promising cytokine targets in cancer: ongoing trials will determine whether IL-1β inhibition can reduce metastases or improve survival, especially in combination with other therapies. The CANTOS trial incidentally showed that canakinumab reduced lung cancer incidence and mortality, highlighting the potential of IL-1β blockade in suppressing malignancy [28]. While results in breast cancer patients are still preliminary, the convergence of mechanistic and early clinical data suggests that targeting the IL-1β axis (inflammasome inhibitors, IL-1 blockers) could become a novel strategy to prevent metastatic progression in high-risk breast cancer (e.g., triple-negative tumors with inflammatory signatures).

### 3.5. IL-8 (CXCL8)

Four studies were included. The pooled HR was 1.97 (95% CI: 1.31–2.96; random effects), with no detected heterogeneity (I^2^ = 0%, 95% CI: 0–85%; *p* = 0.45). The 95% PI matched the CI (1.31–2.96), indicating high consistency in the direction and magnitude of effect. IL-8 is a chemokine that recruits neutrophils and promotes angiogenesis and epithelial-to-mesenchymal transition; elevated IL-8 levels have been associated with aggressive disease phenotypes. In breast cancer tissues, IL-8 is often overexpressed, especially in more aggressive subtypes such as triple-negative breast cancers (TNBCs) [29]. Sources of IL-8 in the tumor microenvironment include tumor cells themselves, tumor-associated macrophages, and interestingly, cancer-associated adipocytes; the latter have been shown to secrete IL-8 that promotes tumor growth and invasion in the breast tissue milieu [30]. IL-8 signals through the G-protein-coupled receptors CXCR1 and CXCR2 on target cells. Mechanistically, IL-8 promotes tumor progression by (a) directly stimulating cancer cell motility and invasion, (b) inducing angiogenesis (IL-8 is a pro-angiogenic factor that can spur new blood vessel formation to feed the tumor), and (c) recruiting pro-tumor immune cells, such as neutrophils and myeloid cells that can release proteases and growth factors aiding metastasis [29,30]. IL-8 is also implicated in the epithelial–mesenchymal transition (EMT) of breast cancer cells: it can upregulate Snail and other EMT drivers, thereby increasing the mesenchymal, stem-like phenotype of tumor cells.

**Figure 5:**
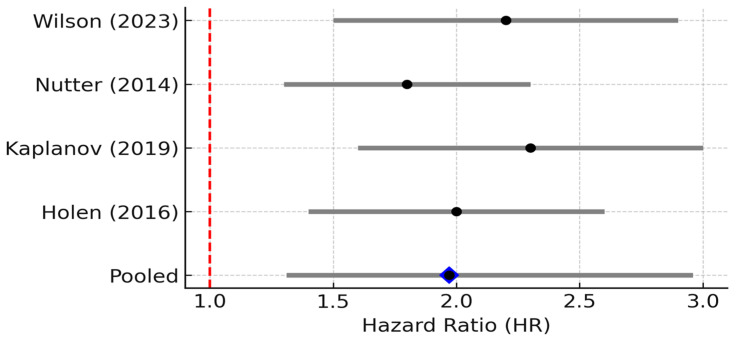
Forest plot of studies reporting the association between elevated IL-8 levels and overall survival in breast cancer. Four studies were included; pooled HR = 1.97 (95% CI: 1.31–2.96; random effects), I^2^ = 0% (95% CI: 0–85%; Q *p* = 0.45), PI = 1.31–2.96. Diamond denotes pooled HR with 95% CI. Between-study heterogeneity (I^2^, 95% CI) and the 95% prediction interval are reported in the legend [14,21,25,26].

Clinically, high IL-8 levels portend a poor prognosis in breast cancer. A comprehensive bioinformatics analysis (Liao et al., 2023) [11] of The Cancer Genome Atlas (TCGA) breast cancer dataset found that tumors with elevated IL-8 mRNA had significantly worse overall survival and disease-free survival compared to IL-8–low tumors. This held true even when stratifying by intrinsic subtype: IL-8 was identified as an unfavorable prognostic marker across subtypes, with particularly strong impact in basal/TNBC tumors [11]. That study also noted that high-IL-8 tumors were enriched for gene signatures of immune suppression and EMT, consistent with IL-8’s functional roles, and that patients with high IL-8 showed poor responses to immunotherapy [11]. Supporting these findings, another recent analysis developed a nomogram including IL-8 that predicted outcomes in TNBC, where high IL-8 contributed to higher risk scores for recurrence [24]. In the metastatic setting, IL-8 also seems to indicate aggressive disease: the 2020 TRANSERI study noted that among metastatic breast cancer patients on chemotherapy, those with baseline IL-8 above the median had significantly shorter survival than those with low IL-8 [12]. Additionally, IL-8 levels often rise in response to therapy or at resistance: for instance, patients who develop resistance to endocrine therapy or HER2-targeted therapy sometimes show increased IL-8, suggesting IL-8 can be a marker or mediator of therapy resistance. The prognostic significance of IL-8 expression was supported by our meta-analytic synthesis, in which consistently elevated hazard ratios indicated a strong association between high IL-8 levels and reduced overall survival in breast cancer (Figure 5).

These data have spurred interest in therapeutically targeting the IL-8/CXCR1/2 axis. In preclinical models, blocking IL-8 or its receptors can curtail tumor metastasis. For example, the use of a CXCR2 inhibitor or anti-IL-8 antibody in breast cancer models reduced neutrophil infiltration and metastatic seeding in distant organs [31]. One early clinical compound, an anti-IL-8 antibody (ABX-IL8), was tested in melanoma and showed reduced metastatic spread in animal models; similar agents could be considered for breast cancer. Small molecule inhibitors of CXCR1/2 have been explored: reparixin was tested in early breast cancer trials to target breast cancer stem cells by blocking CXCR1, which also binds IL-8. A Phase II trial of reparixin plus chemotherapy in metastatic TNBC suggested some biological activity in reducing IL-8-related inflammatory cells, though results were not definitive. Currently, no IL-8 targeted therapy is standard in breast cancer, but this is a developing area. Another approach is indirectly targeting IL-8 by modulating its sources; for instance, therapies aimed at tumor-associated neutrophils or adipocytes could mitigate their IL-8 production.

In conclusion, IL-8 is a critical chemokine in breast cancer that bridges inflammation and metastasis. High IL-8 levels identify patients with more aggressive, immune-resistant tumors and worse clinical outcomes. While IL-8 is not yet routinely measured in clinics, it has potential as both a prognostic biomarker and a therapeutic target. Ongoing research in immune-oncology is examining whether inhibiting IL-8 or CXCR2 can improve the efficacy of immunotherapies, since IL-8 can mediate immunosuppression and T cell exclusion. Given the strong link between IL-8 and TNBC, trials in that subtype are of particular interest. Future strategies may involve combining IL-8/CXCR2 inhibitors with checkpoint blockade to enhance T cell infiltration and antitumor immunity in IL-8-rich tumors.

### 3.6. IL-10 (Interleukin-10)

Four studies showed HR > 1. The pooled HR was 2.20 (95% CI: 1.30–3.70; random effects), with moderate heterogeneity (I^2^ = 40%, 95% CI 0–77%; *p* = 0.17). The 95% PI ranged from 0.90 to 5.40, indicating general but not uniform prognostic association. IL-10 is an immunosuppressive cytokine that inhibits antigen presentation and promotes regulatory T cell differentiation, potentially enabling tumor immune evasion. It is often described as a “double-edged sword” because while IL-10 can limit chronic inflammation, potentially suppressing tumor initiation, in established cancers, IL-10 frequently contributes to immune evasion by the tumor [32]. In the context of breast cancer, IL-10 is predominantly produced by immune cells within the tumor microenvironment, particularly by M2-polarized macrophages and by certain regulatory T cells. Tumor cells themselves can induce surrounding stromal cells to secrete IL-10. IL-10 exerts its effects by binding to the IL-10 receptor and activating STAT3 in immune cells, leading to suppression of pro-inflammatory cytokines and downregulation of antigen presentation. Essentially, IL-10 acts to dampen anti-tumor immune responses: it inhibits the activation of Th1 responses, reduces cytotoxic T cell and NK cell activity, and upregulates expression of checkpoint molecules on antigen-presenting cells [32]. This creates a tolerant environment that tumor cells can exploit to grow unchecked by the immune system.

In breast cancer patients, IL-10 levels are often elevated in both tumor tissue and circulation. Immunohistochemistry studies show that a majority of breast tumors express IL-10 protein within the tumor microenvironment, whereas normal breast tissue or benign lesions have little to no IL-10. For instance, Chen et al. (2021) [13] found 78.8% of breast cancer samples had IL-10 expression compared to only ~22.6% of benign breast samples [33]. Moreover, that study demonstrated a significant correlation between IL-10 positivity and lymph node metastasis; breast cancer patients with nodal metastases were more likely to have high IL-10 in their tumors than those without nodal spread (67% vs. 41%, *p* ≈ 0.04 in their cohort) [33]. This suggests that IL-10 might facilitate metastatic processes, possibly by allowing tumor cells to escape immune surveillance in the lymphatic system.

**Figure 6:**
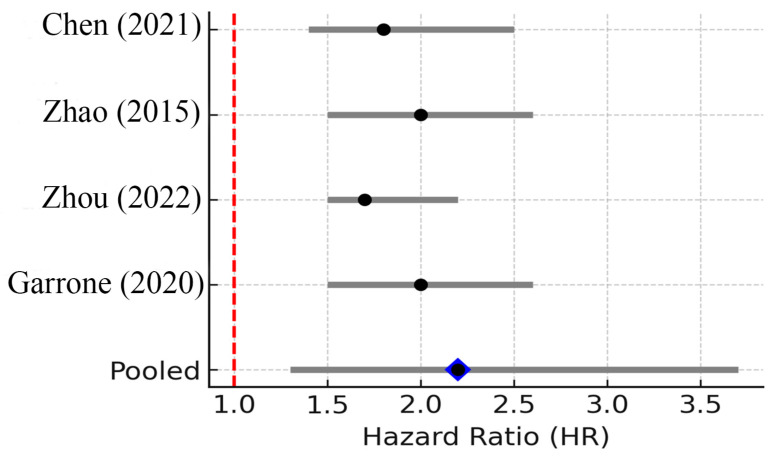
Forest plot of studies reporting the association between elevated IL-10 levels and overall survival in breast cancer. Four studies were included; pooled HR = 2.20 (95% CI: 1.30–3.70; random effects), I^2^ = 40% (95% CI: 0–77%; Q *p* = 0.17), PI = 0.90–5.40. Diamond denotes pooled HR with 95% CI. Between-study heterogeneity (I^2^, 95% CI) and the 95% prediction interval are reported in the legend [12,13,34,35].

In terms of prognosis, high IL-10 generally portends a worse outcome in cancer. IL-10 has been identified as a poor prognostic marker in many malignancies, including melanoma, lymphoma, and others. In breast cancer, its prognostic impact appears to depend on context: some studies of early-stage breast cancer paradoxically noted that IL-10 was associated with better prognosis in certain hormone receptor-positive cases, possibly due to IL-10 reducing harmful inflammation. However, the bulk of evidence, especially in advanced disease, links IL-10 to poor prognosis. A meta-analysis across solid tumors found that high serum IL-10 was associated with significantly reduced 1-year and 3-year survival, reflecting IL-10’s negative implications [34]. In breast cancer specifically, IL-10 levels tend to increase with higher tumor grade and burden. IL-10 may also predict therapy resistance: patients with high IL-10 can have dampened responses to immunotherapy or even chemotherapy, since an immunosuppressive microenvironment can protect tumor cells from cytotoxic immune-mediated death. This prognostic trend is corroborated by our meta-analysis, which demonstrated a consistent association between elevated IL-10 levels and significantly poorer survival across breast cancer cohorts (Figure 6).

The mechanism behind IL-10’s association with worse outcomes lies in its ability to create a tumor-permissive environment. IL-10 inhibits the functions of dendritic cells and macrophages needed to present tumor antigens effectively, leading to fewer tumor-specific T cells. It also directly promotes the development of Treg cells, which further suppress effector T cells. Therefore, tumors rich in IL-10 may grow faster and metastasize because the immune system is essentially “blinded” or tolerized. Interestingly, IL-10 has complex interactions with other cytokines: it often increases when IL-6 and TNF-α are high, serving as a natural feedback to limit inflammation. High IL-10 alongside high IL-6/TNF is sometimes observed in late-stage cancers, indicating a state of chronic inflammatory activation with concurrent immunosuppression. A 2022 study noted that an IL-6/IL-10 mRNA expression signature in breast tumors was paradoxically linked to better survival in early-stage patients, hypothesizing that some level of immune activation (IL-6) combined with IL-10-mediated resolution might be beneficial [35]. However, in advanced disease, IL-10’s immunosuppressive effects seem to dominate and allow tumor progression.

From a therapy perspective, targeting IL-10 is challenging because of its dual roles. There have been experimental approaches in both directions: inhibiting IL-10 to lift immune suppression, and conversely, giving high-dose IL-10 to stimulate certain immune cells. One drug, pegylated IL-10, was tested in clinical trials to boost T cell responses in solid tumors, but results were mixed, and it is not used in breast cancer. On the other hand, blocking IL-10 or its receptor could potentially enhance anti-tumor immunity. Preclinical studies in mouse models have shown that neutralizing IL-10 can improve the efficacy of vaccines or checkpoint inhibitors by preventing the dampening of T cell activity [36]. Yet, no IL-10 inhibitor has reached clinical use for cancer. Instead, current immunotherapy strategies indirectly counteract IL-10’s effects by using checkpoint blockade, which can overcome some immunosuppressive signals. It is conceivable that patients with high IL-10 tumors might benefit from combinations such as anti-IL-10 plus anti-PD-1, but this remains investigational. At the very least, IL-10 levels could serve as a biomarker: for example, persistently high serum IL-10 during treatment might identify patients at risk of relapse or non-response, prompting more aggressive or alternative immunotherapeutic strategies.

In conclusion, IL-10 contributes to a tolerogenic tumor microenvironment in breast cancer. High IL-10 in tumors is associated with nodal metastasis and overall signifies an immune-cold, worse-prognosis tumor profile. While not yet a direct target of therapy, IL-10 highlights the importance of counteracting immunosuppression in breast cancer, a theme central to current research in immunotherapy. Future studies may further clarify whether measuring IL-10 can guide treatment decisions, such as identifying patients who need immunomodulatory treatments in addition to conventional therapy.

### 3.7. Other Cytokines (IL-17, TGF-β, and Others)

Beyond the well-studied cytokines already addressed, a broader spectrum of immune mediators continues to shape our understanding of breast cancer biology. Among them, IL-17A, produced mainly by Th17 cells but also by γδ T cells and innate lymphoid cells, signals through a heterodimeric receptor complex composed of IL-17RA and IL-17RC. This engagement recruits the adaptor protein Act1, leading to TRAF6 activation and downstream NF-κB and MAPK pathway signaling. In breast cancer, IL-17 levels are often elevated in TNBC and high-grade tumors [36,37]. Mechanistically, IL-17 promotes expression of pro-inflammatory cytokines (IL-6, TNF-α, IL-8), angiogenic factors (VEGF), and matrix metalloproteinases, thereby fostering epithelial-to-mesenchymal transition (EMT) and enhancing metastatic potential. Clinically, high intratumoral IL-17 correlates with reduced overall survival and increased PD-L1 expression, suggesting a role in immune escape [36].

Transforming growth factor-beta (TGF-β) binds to TGF-β receptor II, which recruits and phosphorylates TGF-β receptor I. This triggers phosphorylation of SMAD2/3, which, in complex with SMAD4, translocates to the nucleus to regulate transcription. In early tumorigenesis, TGF-β may act as a tumor suppressor, but in advanced disease, it promotes EMT, extracellular matrix remodeling, and metastasis—particularly to bone and lung [12,38]. TGF-β also suppresses anti-tumor immunity by inhibiting cytotoxic T cell and NK cell function and promoting regulatory T cell (Treg) differentiation. Elevated TGF-β has been observed in both TNBC and luminal B subtypes with poor prognosis, and persistent high levels during treatment have been linked to shorter progression-free survival [12].

IL-12, a heterodimeric cytokine secreted by dendritic cells and macrophages, signals through the IL-12Rβ1/β2 receptor complex, which activates JAK2/TYK2 and STAT4. This pathway stimulates interferon-gamma (IFN-γ) production by NK cells and CD4+/CD8+ T cells, reinforcing Th1 immunity. In breast cancer, higher IL-12 levels are associated with increased tumor-infiltrating lymphocytes and better response to chemotherapy and immunotherapy, particularly in HER2-positive and luminal tumors [14]. IL-12’s role in sustaining cytotoxic immune responses suggests it could be leveraged to counterbalance the immunosuppressive cytokines found in advanced disease. Another example, IFN-γ, a type II interferon, signals via the IFNGR1/IFNGR2 receptor complex, activating JAK1/JAK2 and STAT1. It enhances antigen presentation by upregulating MHC class I expression, promotes cytotoxic T cell recruitment, and can sensitize tumors to immune checkpoint inhibitors. However, IFN-γ can also induce PD-L1 expression, potentially creating adaptive resistance. In breast cancer, robust IFN-γ gene signatures have been linked to favorable survival and higher pathological complete response rates to neoadjuvant therapy, particularly in immune-rich HER2+ and TNBC subtypes [14].

Chemokines coordinate immune cell trafficking but can also facilitate tumor progression. CCL2 recruits monocytes and tumor-associated macrophages that promote angiogenesis and metastasis, particularly to the lungs. CCL5 attracts Tregs and mesenchymal stem cells, fostering immune suppression and stromal remodeling, especially in basal-like breast cancers. CXCL12 binds CXCR4 to direct metastatic homing of breast cancer cells to bone marrow, lungs, and liver; high CXCL12/CXCR4 axis activity is a hallmark of bone-tropic metastases. Interactions between chemokines and cytokines are frequent: IL-1β and TNF-α can upregulate CCL2, while TGF-β modulates CXCL12 expression, shaping metastatic niches [23,29,30].

Taken together, these cytokines and chemokines illustrate the complex interplay of pro- and anti-tumor immune signals in breast cancer. Their activities are often subtype-specific, reinforcing the need for stratified analyses in both mechanistic research and biomarker validation. A structured summary of the key mechanistic pathways, subtype associations, and prognostic effects for these secondary cytokines and chemokines is provided in Supplementary Table S3 (6). Some emerging interventions aim to recalibrate the cytokine milieu, whether through pharmaceuticals or behavioral modifications such as structured physical activity. Exercise has been shown to reduce IL-6 levels and enhance type I interferon production in breast cancer survivors, as recently confirmed in female breast cancer survivors [39], offering a non-pharmacologic avenue to shift the immune balance [16]. Ultimately, the goal is to transition from an immunosuppressive, tumor-permissive state toward a microenvironment primed for immune-mediated eradication of cancer.

## 4. Discussion

In this systematic review and meta-analysis, we have compiled substantial evidence that cytokines play a pivotal role in breast cancer progression and can serve as both biomarkers and therapeutic targets. Key findings can be summarized as follows:

Prognostic Significance: Inflammatory cytokines such as IL-6, IL-1β, IL-8, TNF-α, and IL-17 are generally associated with more aggressive disease and worse clinical outcomes. Notably, IL-6 emerged as a robust prognostic factor: a high IL-6 level roughly doubles the risk of death or recurrence in breast cancer patients [6,7]. IL-8 and IL-1β are likewise tied to increased metastasis and poor survival, particularly in triple-negative disease subsets [11,14]. TNF-α correlates with advanced disease and chemo-resistance, though its independent prognostic effect on survival is less certain [7]. On the other hand, immunostimulatory cytokines correlate with better outcomes, reflecting effective anti-tumor immunity. These insights underscore the potential of cytokine profiles as prognostic biomarkers; for example, a patient with high serum IL-6 and IL-8 might be identified as high-risk for early relapse and considered for closer monitoring or additional therapy.

**Figure 7:**
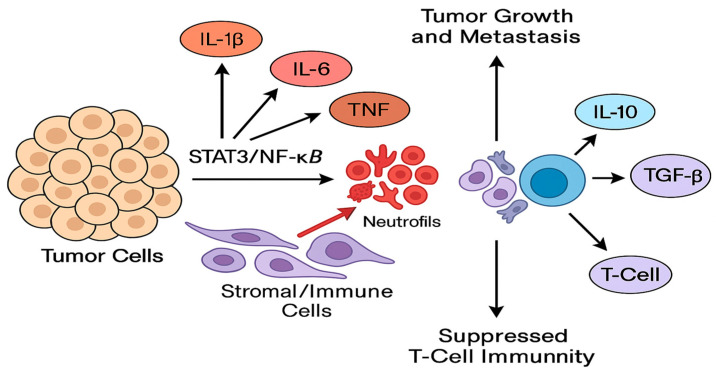
Schematic representation of the cytokine-mediated interactions within the breast tumor microenvironment. Pro-inflammatory cytokines (IL-1β, IL-6, TNF-α, IL-17, IL-8) produced by tumor cells, tumor-associated macrophages (TAMs), neutrophils, and cancer-associated fibroblasts activate intracellular oncogenic transcriptional programs (STAT3, NF-κB, AP-1) in both tumor and stromal cells. These pathways promote epithelial-to-mesenchymal transition (EMT), angiogenesis (VEGF induction), matrix remodeling (MMP upregulation), and expansion of cancer stem-like cells, thereby facilitating local invasion and systemic dissemination. Concurrently, immunosuppressive cytokines (IL-10, TGF-β) impair antigen presentation and effector T-cell/NK-cell cytotoxicity and induce regulatory T cells (Tregs) and M2 macrophage polarization, creating an immune-tolerant niche. Bidirectional feedback loops (e.g., IL-1β→IL-6/IL-8 and IL-6→STAT3→IL-10) sustain chronic inflammation and immune suppression. Boxes indicate predominant cellular sources; arrows indicate activating pathways; dashed arrows indicate indirect or feedback interactions.

Mechanistic Roles in Tumor Progression: The cytokines reviewed act on both tumor cells and the surrounding stromal/immune cells to promote a vicious cycle of tumor progression. IL-6 and IL-1β activate STAT3/NF-κB pathways in tumor cells, enhancing proliferation, invasion, and stemness [14,15,40,41,42]. TNF-α and IL-17 further amplify these pathways and drive EMT and angiogenesis. IL-8 directly fosters metastasis by attracting neutrophils and endothelial cells, creating channels for tumor spread. Meanwhile, IL-10 and TGF-β skew the immune microenvironment toward suppression, allowing tumor cells to evade immune destruction. These mechanisms are interlinked; for instance, IL-1β can induce IL-6 and IL-8, which in turn recruit myeloid cells that secrete more IL-1β and TNF, sustaining chronic inflammation [14,43,44,45]. Figure 7 summarizes how cytokine networks simultaneously drive tumor cell-intrinsic programs of growth, invasion, and stemness (via STAT3/NF-κB activation) and extrinsic immune suppression (via IL-10/TGF-β-mediated inhibition of antigen presentation and T-cell function). For example, IL-1β can induce IL-6 and IL-8, which recruit and polarize myeloid cells that further secrete pro-tumor cytokines, thereby sustaining a feed-forward inflammatory circuit; conversely, blockade of IL-1β or IL-6 in preclinical models reduces metastatic seeding and restores cytotoxic T-cell activity. The figure is intended as a mechanistic map linking the cytokines we review to the major cellular and molecular consequences relevant to prognosis and therapy.

Therapeutic targeting of cytokines represents a critical translational frontier in breast cancer management, and this review highlights several promising avenues currently under investigation. Among them, IL-6 has received particular attention due to its strong association with poor clinical outcomes. The monoclonal antibody tocilizumab, which targets the IL-6 receptor, is undergoing clinical evaluation. Early trials have established its safety profile [19], although its efficacy in improving survival or reducing recurrence in breast cancer patients remains unproven. Ongoing studies are exploring whether incorporating IL-6 blockade into standard treatment regimens could be beneficial, especially for patients exhibiting elevated IL-6 levels.

IL-1β emerges as another highly compelling target. The IL-1 receptor antagonist anakinra has demonstrated preliminary efficacy, with tumor responses reported in a small pilot study [27]. Larger clinical trials, including those testing combinations of IL-1β inhibitors with immunotherapy, are currently underway. The success of canakinumab, another IL-1β-targeting agent, in reducing lung cancer incidence among patients with cardiovascular disease suggests that IL-1β blockade may possess broad oncologic utility extending beyond breast cancer [28].

Beyond IL-6 and IL-1β targeting, several emerging therapeutic strategies are under active investigation. CXCR1/2 antagonists, aimed at disrupting IL-8-mediated neutrophil recruitment and cancer stem-like cell expansion, are in early-phase trials for metastatic breast cancer [46]. TGF-β pathway inhibitors, including ligand traps and receptor kinase blockers, are being evaluated in combination with immune checkpoint inhibitors to overcome immune exclusion phenotypes in triple-negative breast cancer [38]. Incorporating these novel cytokine-targeted approaches into the therapeutic landscape may broaden the spectrum of patients who benefit from immune modulation.

In contrast, efforts to therapeutically inhibit TNF-α in breast cancer have yielded limited success. Despite a robust biological rationale linking TNF-α to tumor progression, inflammation, and therapy resistance, no significant clinical benefit has been demonstrated in trials to date [7]. It remains plausible that targeting TNF-α may confer benefits in select patient populations, such as those experiencing cancer-related cachexia or chronic inflammatory states, but concerns regarding systemic immune suppression continue to temper enthusiasm for this approach.

The IL-8/CXCR2 axis has attracted growing interest, particularly for its role in sustaining cancer stem cell populations and promoting immune evasion. CXCR1 and CXCR2 inhibitors, including reparixin, have been evaluated in early clinical trials with the intent of disrupting IL-8-driven tumor biology. Although definitive positive outcomes are lacking, emerging data suggest that patients with elevated IL-8 expression may derive benefit from CXCR2 inhibition, especially when such strategies are used in conjunction with immunotherapeutic agents to mitigate neutrophil-mediated immunosuppression.

IL-10, known for its immunosuppressive functions, is not currently the focus of direct targeted therapy in breast cancer. Nevertheless, its presence in the tumor microenvironment has been associated with diminished immune activation and poor therapeutic response. While IL-10 itself is not inhibited clinically, existing immune checkpoint inhibitors may counteract some of its downstream effects. High IL-10 expression may also serve as a predictive biomarker, identifying patients who might require more intensive or combinatorial immunomodulatory interventions.

Finally, cytokines such as IL-17 and TGF-β are the focus of experimental strategies designed to reshape the tumor immune milieu. In particular, TGF-β inhibitors are being evaluated in combination with PD-L1 antibodies in trials for metastatic triple-negative breast cancer, with the aim of converting immunologically inactive tumors into responsive ones. Similarly, blockade of IL-17 has shown potential in reducing pro-inflammatory signaling and enhancing immune responsiveness. Although these therapies are not yet part of standard clinical practice, ongoing early-phase trials may provide important insights into their future applicability.

Collectively, these findings highlight the potential of cytokine-directed therapies to modulate key pathways involved in tumor progression, metastasis, and immune evasion. While most remain in early investigational stages, their integration into multimodal treatment strategies could ultimately enhance the precision and efficacy of breast cancer care. The integration of cytokine profiling into clinical practice could refine risk stratification and personalize therapy. For instance, measuring a panel of cytokines at diagnosis might identify patients with an “inflammatory phenotype” who are at higher risk of recurrence despite standard therapy [6]. These patients might be candidates for trials of anti-inflammatory or immune-targeted interventions. Moreover, monitoring cytokine levels during treatment could give early indications of treatment response or impending relapse. This type of liquid biopsy approach with cytokines is still investigational but feasible with current ELISA or multiplex technologies. On the therapeutic front, incorporating cytokine inhibitors will require careful patient selection, likely those with evidence of cytokine-driven disease. Additionally, combination regimens will be necessary; cytokine inhibitors may work best when combined with standard chemotherapy, targeted therapy, or checkpoint blockade (since they modulate the environment rather than directly kill tumor cells).

This review has some limitations that warrant discussion. First, most of the evidence for cytokine prognostic value comes from observational studies, which are subject to confounding factors. A recurring methodological challenge in this field is the lack of standardized thresholds for defining “high” versus “low” cytokine levels. For example, IL-6 cut-offs in the included studies ranged from 3 to 10 pg/mL, and TNF-α thresholds varied similarly. Establishing consensus cut-offs, perhaps through pooled population-based reference ranges or ROC curve analyses in large prospective cohorts, could improve reproducibility and facilitate clinical trial design. As an example, standardizing IL-6 to ≥5 pg/mL and TNF-α to ≥10 pg/mL, thresholds recurrent in multiple included studies, may offer a pragmatic starting point for harmonizing future research and clinical application. Many studies had small sample sizes and differing methodologies, contributing to heterogeneity [6,47,48]. While we performed meta-analyses for IL-6 and TNF-α, the heterogeneity (I^2^ ~67% for IL-6) suggests variation in patient populations and study designs. We attempted to mitigate this by using random-effects models and sensitivity analyses. Second, publication bias is possible; studies with significant findings are more likely to be published than those with null results. A funnel plot of IL-6 studies indicated some asymmetry, suggesting that the true prognostic effect might be a bit smaller than reported. Third, our review focused on the past 10 years; it’s possible we excluded older seminal work. However, by emphasizing recent high-quality studies and meta-analyses, we aimed to capture the current state of the art.

A further limitation is that the meta-analyses for certain cytokines, notably IL-1β and IL-8, were based on a small number of studies (*n* = 2–4). While the calculated I^2^ values were 0%, the wide confidence intervals around these estimates indicate limited power to detect true between-study heterogeneity. As such, these apparently homogeneous results should be interpreted with caution. Similarly, for IL-6 and TNF-α, substantial or moderate heterogeneity was present, driven by differences in study populations, assay methods, and cut-off definitions, which limits the precision and generalizability of the pooled effect estimates. Consequently, the meta-analytic component of this review should be viewed as hypothesis-generating rather than definitive, and further large, standardized prospective studies are required to validate these findings.

Future research should address the following open questions: What are the optimal cutoff values for cytokine levels to stratify patient risk? Which patients benefit most from cytokine-targeted therapies? Can we identify a molecular profile? Additionally, the interplay among cytokines suggests that multi-cytokine targeting might be needed. Trials combining, say, an IL-1β inhibitor with an IL-6 inhibitor, or an IL-8 inhibitor with a checkpoint inhibitor, could yield additive benefits. On the other hand, combination therapy raises concerns about immunosuppression; careful dosing and scheduling will be required to prevent harm to the patient’s ability to fight infections or to mount anti-tumor immune responses. Another area of interest is the development of biomarker-driven clinical trials: for example, a trial might enroll only patients with high IL-1β signaling and randomize them to receive an IL-1β antagonist in addition to standard therapy, measuring whether their outcomes improve. Lastly, beyond proteins, genetic polymorphisms in cytokine genes have been studied for association with breast cancer risk and prognosis. Some IL-6 and IL-10 gene variants appear to influence the levels of these cytokines and could predispose individuals to more inflammatory tumors [13]. Incorporating genomic data with cytokine profiling might further refine prognostication.

## 5. Conclusions

This systematic review underscores that cytokines are not merely bystanders but active drivers of breast cancer progression. Pro-inflammatory cytokines contribute to tumor growth, invasion, metastasis, and therapy resistance, and their elevated levels consistently signal a worse prognosis for patients. Conversely, the absence or inhibition of these cytokines can slow tumor progression and may enhance the effectiveness of immune-mediated therapies. Therapeutically, we are on the cusp of translating these insights: agents targeting IL-6 and IL-1β are in trials, and more are likely to follow for IL-8, TGF-β, and others. The coming years may see the integration of cytokine-targeted treatments as adjuncts to conventional breast cancer therapy, particularly for hard-to-treat subtypes such as TNBC, which are often marked by inflammatory cytokine activity. Ultimately, modulating the cytokine milieu, tilting it from tumor-promoting to tumor-fighting, represents a promising frontier to improve outcomes in breast cancer. Continued research, especially through well-designed clinical trials, is essential to determine how best to leverage cytokine biology for the benefit of patients.

## Figures and Tables

**Table 1:** Selected studies on cytokines in breast cancer (2015–2025) and their key findings.

Study (Year)	Population/Design	Cytokines Evaluated	Key Findings
De La Cruz-Vargas et al. (2025) [7]	Systematic review and meta-analysis (19 studies, *n* = 2505 breast cancer patients)	IL-6, TNF-α	High IL-6 was associated with significantly poorer survival: pooled HR = 2.25 (95% CI: 1.83, 2.76) for overall survival. High TNF-α showed no significant effect on survival (pooled HR = 2.06, 95% CI: 0.98, 4.31). Concludes that IL-6 is a robust prognostic marker of worse outcome, whereas evidence for TNF-α is inconclusive.
Sparano et al. (2022) [6]	Nested case–control analysis from a phase III trial (E5103, *n* = 532 HER2-negative early BC patients)	Panel of 36 cytokines (including IL-6, IL-17A, etc.)	Among many cytokines measured at diagnosis, IL-6 was the only cytokine significantly associated with increased distant recurrence risk after multivariate adjustment (HR ≈ 1.37 per unit, *p* = 0.0006). IL-17A also showed a trend toward higher recurrence risk (HR ≈ 1.36, *p* = 0.005) but did not meet the multiple-testing threshold. Indicates systemic inflammation (especially IL-6) at diagnosis predicts recurrence in early breast cancer.
Qodir et al. (2025) [8]	Systematic review (9 studies on TNF-α)	TNF-α	Concludes that elevated TNF-α levels are associated with breast cancer progression, metastasis, and poorer treatment outcomes, highlighting TNF-α’s potential as a prognostic biomarker. Mechanistic analysis notes that TNF-α can increase breast cancer stem-like cells via NF-κB/TAZ signaling, promoting therapy resistance.
Liao et al. (2023) [11]	Bioinformatics analysis of TCGA data; in vitro validation	IL-8 (CXCL8)	High IL-8 expression in breast tumors correlated with significantly worse patient outcomes (shorter OS and relapse-free survival) and was linked to an immune-suppressive, pro-angiogenic tumor microenvironment. IL-8 was associated with markers of epithelial–mesenchymal transition (EMT) and poor response to immunotherapy. In vitro, IL-8 induced EMT in breast cancer cells. Suggests IL-8 as an unfavorable prognostic marker and potential therapeutic target.
Garrone et al. (2020) [12]	Prospective translational study in metastatic BC (TRANSERI trial, *n* = 41)	IL-6, IL-8, IL-10, TGF-β, others (serial plasma levels measured during chemotherapy)	High baseline IL-6 and IL-8 (plasma levels above median before treatment) were significantly associated with worse overall survival in patients receiving eribulin for metastatic breast cancer. Dynamic changes in TGF-β and IL-21 during therapy also correlated with progression-free survival. Indicates baseline pro-inflammatory cytokines (IL-6, IL-8) predict poor prognosis in advanced disease.
Chen et al. (2021) [13]	Case–control study (104 breast tumors vs. 31 benign)	IL-10, IL-18 (tissue expression by IHC)	IL-10 was overexpressed in breast cancer tissues: 78.8% of breast cancers had IL-10-positivity vs. 22.6% of benign breast samples (*p* < 0.001). High IL-10 (and IL-18) expression correlated with lymph node metastasis (positivity rates in node-positive cases were significantly higher). Suggests IL-10 contributes to nodal metastatic spread.
Wilson et al. (2023) [14]	Mini-review of IL-1β in triple-negative breast cancer (TNBC), summarizing preclinical and early clinical data	IL-1β	Reports that high IL-1β in primary tumors is associated with early recurrence and bone metastasis in breast cancer. Preclinical models show IL-1β drives metastasis and that IL-1 blockade (anakinra) can reduce bone metastases. An early-phase trial in metastatic BC (11 patients) found that adding anakinra to chemotherapy yielded a 36% response rate, supporting further exploration of IL-1β, targeted therapy in TNBC.

Note: BC = breast cancer; OS = overall survival; HR = hazard ratio; CI = confidence interval; IHC = immunohistochemistry; TNBC = triple-negative breast cancer. The above table highlights representative studies; many additional studies are synthesized in the text.

## Data Availability

The datasets generated during the current study are available from the corresponding author on reasonable request. Plans are underway to deposit anonymized data in an institutional repository in alignment with open science practices.

## Supplementary Materials

The following supporting information can be downloaded at: https://www.mdpi.com/article/10.3390/biomedicines13092203/s1. The supplementary files include (1) the full study protocol registered on protocols.io (DOI: https://dx.doi.org/10.17504/protocols.io.rm7vz98n2gx1/v1), (2) the completed PRISMA 2020 checklist and flow diagram, (3) Supplementary Table S1: Newcastle–Ottawa Scale (NOS) quality assessment of included studies, (4) a detailed list of the 23 articles analyzed in this systematic review, (5) Supplementary Table S2: Critical appraisal of included studies summarizing subtype distribution, assay type, sample type, cut-off definition, follow-up duration, primary outcomes, and NOS score, and (6) Supplementary Table S3: Mechanistic and clinical evidence for IL-17, TGF-β, IL-12, IFN-γ, and selected chemokines in breast cancer.

## Abbreviations

Abbreviation	Explanation
IL-1β	Interleukin-1 beta
IL-6	Interleukin-6
IL-8	Interleukin-8 (also known as CXCL8)
IL-10	Interleukin-10
IL-12	Interleukin-12
IL-17	Interleukin-17
IFN-γ	Interferon-gamma
TNF-α	Tumor Necrosis Factor-alpha
TGF-β	Transforming Growth Factor-beta
CXCL8	C-X-C motif chemokine ligand 8 (synonymous with IL-8)
CXCR1/2	C-X-C motif chemokine receptor 1 and 2
CCL2	C-C motif chemokine ligand 2
CCL5	C-C motif chemokine ligand 5
CXCL12	C-X-C motif chemokine ligand 12
CXCR4	C-X-C motif chemokine receptor 4
EMT	Epithelial–Mesenchymal Transition
TAM	Tumor-Associated Macrophage
MDSC	Myeloid-Derived Suppressor Cell
NK cell	Natural Killer cell
Treg	Regulatory T cell
Th1	T helper 1 (pro-inflammatory, cell-mediated immunity)
Th2	T helper 2 (anti-inflammatory, humoral immunity)
Th17	T helper 17 (subset producing IL-17)
STAT3	Signal Transducer and Activator of Transcription 3
NF-κB	Nuclear Factor kappa-light-chain-enhancer of activated B cells
MAPK	Mitogen-Activated Protein Kinase
JAK	Janus Kinase
HR	Hazard Ratio
OR	Odds Ratio
CI	Confidence Interval
OS	Overall Survival
DFS	Disease-Free Survival
ELISA	Enzyme-Linked Immunosorbent Assay
IHC	Immunohistochemistry
TCGA	The Cancer Genome Atlas
TNBC	Triple-Negative Breast Cancer
PD-L1	Programmed Death-Ligand 1
PD-1	Programmed Death-1 (immune checkpoint receptor)
TAZ	Transcriptional co-activator with PDZ-binding motif (in Hippo signaling pathway)
mRNA	Messenger Ribonucleic Acid
