# Supplementary material for: Role of Cytokines in Breast Cancer: A Systematic Review and Meta-Analysis
Source: Biomedicines. 2025 Sep 9;13(9):2203. doi: 10.3390/biomedicines13092203 (PMC12467893; doi:10.3390/biomedicines13092203)
Supplement: Supplementary file 1 [file biomedicines-13-02203-s001.zip › Articles_List.pdf]

List of Articles analysed and Links

Title	Link/DOI
Contribution of Interleukin-10 Genotype to Triple Negative Breast Cancer Risk	https://ar.iiarjournals.org/content/41/5/2451
Circulating Cytokines in Metastatic Breast Cancer Patients Select Different Prognostic Groups and Patients Who Might Benefit from Treatment beyond Progression	https://www.mdpi.com/2076-393X/10/1/78/htm
Effects of exercise on inflammation in female survivors of nonmetastatic breast cancer: a systematic review and meta-analysis	https://dx.doi.org/10.1093/jnci/djaf062
Inflammatory cytokines and distant recurrence in HER2-negative early breast cancer	https://www.nature.com/articles/s41523-021-00376-9
The role of Th-17 cells and IL-17 in the metastatic spread of breast cancer: As a means of prognosis and therapeutic target	https://pmc.ncbi.nlm.nih.gov/articles/PMC10040566/
The potential role and status of IL-17 family cytokines in breast cancer	https://pubmed.ncbi.nlm.nih.gov/33740640/
IL-6/IL-10 mRNA expression ratio in tumor tissues predicts prognosis in gastric cancer patients without distant metastasis	https://www.nature.com/articles/s41598-022-24189-3
IL-6 and IL-10 are associated with good prognosis in early stage invasive breast cancer patients	https://link.springer.com/article/10.1007/s00262-017-2106-8
Serum IL-10 Predicts Worse Outcome in Cancer Patients: A Meta-Analysis	https://journals.plos.org/plosone/article?id=10.1371/journal.pone.0139598
The role of IL-8 in cancer development and its impact on immunotherapy resistance	10.1016/J.EJCA.2025.115267
High CXCL8 expression predicting poor prognosis in triple-negative breast cancer	https://pmc.ncbi.nlm.nih.gov/articles/PMC11781556/
Targeting cancer-associated adipocyte-derived CXCL8 inhibits triple-negative breast cancer progression and enhances the efficacy of anti-PD-1 immunotherapy	https://www.nature.com/articles/s41419-023-06230-z
Inhibition of Interleukin-8/C-X-C Chemokine Receptor 2 Signaling Axis Prevents Tumor Growth and Metastasis in Triple-Negative Breast	https://pmc.ncbi.nlm.nih.gov/articles/PMC12105825/

Cancer Cells	
Cytokines as Mediators of Pain-Related Process in Breast Cancer	https://onlinelibrary.wiley.com/doi/full/10.1155/2015/129034
Exploring immune interactions in triple negative breast cancer: IL-1β inhibition and its therapeutic potential	https://www.frontiersin.org/journals/genetics/articles/10.3389/fgene.2023.1086163/full
Interleukin-18 and −10 may be associated with lymph node metastasis in breast cancer	https://pmc.ncbi.nlm.nih.gov/articles/PMC7882877/
Exploratory analysis of circulating cytokines in patients with metastatic breast cancer treated with eribulin: the TRANSERI-GONO (Gruppo Oncologico del Nord Ovest) study	https://pubmed.ncbi.nlm.nih.gov/33051191/
Expression of the prognostic marker IL-8 correlates with the immune signature and epithelial-mesenchymal transition in breast cancer	https://pubmed.ncbi.nlm.nih.gov/36725216/
Inflammatory cytokines and distant recurrence in HER2-negative early breast cancer	https://www.nature.com/articles/s41523-021-00376-9
Prognostic Relevance of Inflammatory Cytokines Il-6 and TNF-Alpha in Patients with Breast Cancer: A Systematic Review and Meta-Analysis	https://pmc.ncbi.nlm.nih.gov/articles/PMC12192186/
Tumor Necrosis Factor-Alpha and Its Association With Breast Cancer: A Systematic Review	https://wjon.elmerpub.com/wjon/article/view/2532/123
Interleukin-10: A double-edged sword in breast cancer	https://pmc.ncbi.nlm.nih.gov/articles/PMC8323643/
IL-6: The Link Between Inflammation, Immunity and Breast Cancer	www.frontiersin.org
