# Supplementary material for: Role of Cytokines in Breast Cancer: A Systematic Review and Meta-Analysis
Source: Biomedicines. 2025 Sep 9;13(9):2203. doi: 10.3390/biomedicines13092203 (PMC12467893; doi:10.3390/biomedicines13092203)
Supplement: Supplementary file 1 [file biomedicines-13-02203-s001.zip › Protocol.io DOI link.pdf]

DOI: dx.doi.org/10.17504/protocols.io.rm7vz98n2gx1/v1 (Private link for reviewers: https://www.protocols.io/private/3A9D5BD05F1E11F0B3630A58A9FEAC02 to be removed before publication.)
