# Supplementary material for: Role of Cytokines in Breast Cancer: A Systematic Review and Meta-Analysis
Source: Biomedicines. 2025 Sep 9;13(9):2203. doi: 10.3390/biomedicines13092203 (PMC12467893; doi:10.3390/biomedicines13092203)
Supplement: Supplementary file 1 [file biomedicines-13-02203-s001.zip › Supplementary Table S1 - Newcastle–Ottawa Scale (NOS).pdf]

Study (First Author, Year)	Study Design	Selection (0–4)	Comparability (0–2)	Outcome/Exposure (0–3)	Total Score (0–9)	Quality Category
Chen et al., 2022 (IL-6) – "IL-6: The Link Between Inflammation, Immunity and Breast Cancer"	Cohort	4	2	3	9	High
De La Cruz-Vargas et al., 2025 – "Prognostic Relevance of Inflammatory Cytokines IL-6 and TNF-Alpha"	Systematic review/meta-analysis (clinical data)	4	2	3	9	High
Qodir et al., 2025 – "TNF-Alpha and Its Association with Breast Cancer"	Systematic review (clinical data)	4	2	3	9	High
Sparano et al., 2022 – "Inflammatory Cytokines and Distant Recurrence in HER2-negative Early Breast Cancer"	Nested case-control	4	2	3	9	High
Garrone et al., 2020 – "TRANSERI-GONO Study"	Prospective cohort	4	2	3	9	High
Liao et al., 2023 – "Expression of IL-8 and EMT in Breast Cancer"	Cohort (bioinformatics + validation)	3	2	3	8	High
Tang et al., 2025 – "High CXCL8 Expression in TNBC"	Retrospective cohort	3	1	3	7	High

Study (First Author, Year)	Study Design	Selection (0–4)	Comparability (0–2)	Outcome/Exposure (0–3)	Total Score (0–9)	Quality Category
Huang et al., 2023 – "Adipocyte-derived CXCL8 in TNBC"	Translational cohort	4	2	3	9	High
Jeong et al., 2025 – "Inhibition of IL-8/CXCR2 Axis in TNBC"	Translational cohort	3	2	3	8	High
Wang et al., 2021 – "Interleukin-18 and −10 in Breast Cancer"	Case-control	4	1	3	8	High
Ma et al., 2017 – "IL-6, IL-8, and TNF-α Levels Correlate with Disease Stage"	Case-control	3	1	3	7	High
Fontvieille et al., 2022 – "Inflammatory Biomarkers and Breast Cancer Risk"	Case-control	4	2	3	9	High
Chang et al., 2021 – "Interleukin-10: A Double-Edged Sword in Breast Cancer"	Narrative review + clinical correlation	3	1	2	6	Moderate
Zhao et al., 2015 – "Serum IL-10 Predicts Worse Outcome"	Meta-analysis (clinical data)	4	2	3	9	High
Zhou et al., 2022 – "IL-6/IL-10 mRNA Ratio and Prognosis"	Retrospective cohort	3	1	3	7	High
Bettariga et al., 2025 – "Exercise	Systematic review/meta-	4	2	3	9	High

Study (First Author, Year)	Study Design	Selection (0–4)	Comparability (0–2)	Outcome/Exposure (0–3)	Total Score (0–9)	Quality Category
and Inflammation in Breast Cancer Survivors”	analysis (clinical)					
Shibabaw et al., 2023 – “Th17 Cells and IL-17 in Breast Cancer”	Review + clinical evidence	3	1	2	6	Moderate
Song et al., 2021 – “IL-17 Family Cytokines in Breast Cancer”	Review + clinical evidence	3	1	2	6	Moderate
Panis & Pavanelli, 2015 – “Cytokines as Mediators of Pain in Breast Cancer”	Narrative review + clinical context	3	1	2	6	Moderate
Wilson et al., 2023 – “IL-1β Inhibition in TNBC”	Review + early clinical trial	3	1	2	6	Moderate
Ma et al., 2017 – “IL-6 and IL-10 Associated with Good Prognosis in Early BC”	Cohort	3	2	3	8	High
Chen et al., 2021 – “Contribution of Interleukin-10 Genotype to TNBC Risk”	Case-control	4	1	3	8	High
Meier & Brieger, 2025 – “The Role of IL-8 in Cancer Development”	Review + clinical context	3	1	2	6	Moderate
