# Supplementary material for: Role of Cytokines in Breast Cancer: A Systematic Review and Meta-Analysis
Source: Biomedicines. 2025 Sep 9;13(9):2203. doi: 10.3390/biomedicines13092203 (PMC12467893; doi:10.3390/biomedicines13092203)
Supplement: Supplementary file 1 [file biomedicines-13-02203-s001.zip › Supplementary Table S2 - Critical Appraisal.pdf]

Study (First Author, Year)	Cytokine(s)	Subtype Distribution	Assay Type	Sample Type	Cut-off Definition	Follow-up Duration	Primary Outcome(s)	NOS Score
Chen, 2022	IL-6	Mixed; not reported by subtype	Multiplex bead assay	Plasma	Median	NR	OS, DFS	9
De La Cruz-Vargas, 2025	IL-6, TNF-α	Mixed	ELISA	Serum	ROC-derived	60 mo	OS	9
Qodir, 2025	TNF-α	Mixed	ELISA	Serum	Median	NR	OS, DFS	9
Sparano, 2022	IL-6, IL-17A	HER2– early BC	Multiplex bead assay	Plasma	Median	7.3 y	Distant recurrence	9
Garrone, 2020	IL-6, IL-8, IL-10, TGF-β	Metastatic BC	ELISA	Plasma	Median	24 mo	OS, PFS	9
Liao, 2023	IL-8	Mixed	RNA-seq + qPCR	Tumor tissue	Median	NR	OS	8
Tang, 2025	CXCL8	TNBC	IHC	Tumor tissue	≥10% positive cells	NR	OS	7
Huang, 2023	CXCL8	TNBC	ELISA	Plasma	ROC-derived	NR	OS, PFS	9
Jeong, 2025	CXCL8, CXCR2	TNBC	IHC, WB	Tumor tissue	≥10% positive cells	NR	OS, PFS	8
Wang, 2021	IL-10, IL-18	Mixed	IHC	Tumor tissue	≥10% positive cells	NR	Lymph node metastasis	8
Ma, 2017	IL-6, IL-8, TNF-α	Mixed	ELISA	Serum	Median	NR	OS	7
Fontvieille, 2022	IL-6, IL-8, IL-10	Mixed	ELISA	Plasma	Median	NR	OS	9
Chang, 2021	IL-10	Mixed	IHC	Tumor tissue	NR	NR	OS	6
Zhao, 2015	IL-10	Mixed	ELISA	Serum	Median	NR	OS	9

Study (First Author, Year)	Cytokine(s)	Subtype Distribution	Assay Type	Sample Type	Cut-off Definition	Follow-up Duration	Primary Outcome(s)	NOS Score
Zhou, 2022	IL-6/IL-10 ratio	Mixed	RT-qPCR	Tumor tissue	Median	NR	OS	7
Bettariga, 2025	Multiple cytokines	Mixed	ELISA	Serum	NR	NR	OS	9
Shibabaw, 2023	IL-17	Mixed	IHC	Tumor tissue	NR	NR	OS	6
Song, 2021	IL-17 family	Mixed	ELISA, qPCR	Serum, tumor tissue	NR	NR	OS	6
Panis, 2015	Multiple cytokines	Mixed	ELISA	Serum	NR	NR	Pain-related outcomes	6
Wilson, 2023	IL-1β	TNBC	NR	NR	NR	NR	OS	6
Ma, 2017b	IL-6, IL-10	Early BC	ELISA	Serum	Median	NR	OS	8
Chen, 2021	IL-10 genotype	TNBC	PCR	Blood	NR	NR	Risk association	8
Meier, 2025	IL-8	Mixed	ELISA	Serum	Median	NR	OS	6
