# Supplementary material for: Role of Cytokines in Breast Cancer: A Systematic Review and Meta-Analysis
Source: Biomedicines. 2025 Sep 9;13(9):2203. doi: 10.3390/biomedicines13092203 (PMC12467893; doi:10.3390/biomedicines13092203)
Supplement: Supplementary file 1 [file biomedicines-13-02203-s001.zip › Supplementary Table S3 - Mechanistic and clinical evidence.pdf]

Supplementary Table. Mechanistic and Clinical Evidence for Secondary Cytokines and Selected Chemokines in Breast Cancer

(↑ = increased; ↓ = decreased; OS = overall survival; PFS = progression-free survival; EMT = epithelial–mesenchymal transition)

Cytokine / Chemokine	Main Signaling Pathway	Breast Cancer Subtypes Most Affected	Key Mechanistic Effects	Prognostic Associations	Key References
IL-17A	IL-17RA/RC → Act1 → TRAF6 → NF-κB, MAPK	TNBC, HER2+	Induces IL-6, IL-8, TNF-α; ↑ VEGF; ↑ MMPs; promotes EMT and metastasis	↑ Metastasis; ↓ OS; ↑ PD-L1 expression	[35,36]
TGF-β	TGFBRII → TGFBRI → SMAD2/3 → SMAD4	TNBC, Luminal B	Early stage: tumor suppression; Late stage: ↑ EMT, ↑ ECM remodeling; immune suppression (↑ Tregs, ↓ CTLs)	↑ Metastasis; ↓ PFS/OS; immune exclusion phenotype	[29,45]
IL-12	IL-12Rβ1/β2 → JAK2/TYK2 → STAT4	HER2+, Luminal	↑ Th1 immunity; ↑ IFN-γ production; ↑ CTL and NK activity	↑ OS; ↑ therapy response in immune-rich tumors	[16]
IFN-γ	IFNGR1/2 → JAK1/JAK2 → STAT1	TNBC, HER2+	↑ Antigen presentation (↑ MHC-I); ↑ T cell recruitment; can ↑ PD-L1 (adaptive resistance)	↑ OS; ↑ pCR rates to neoadjuvant therapy	[16]
CCL2 (MCP-1)	CCR2 (G-protein coupled)	Basal-like, TNBC	Recruits monocytes/TAMs; promotes angiogenesis and metastasis	↑ Lung metastasis; ↓ OS	[20]
CCL5 (RANTES)	CCR5	Basal-like	Recruits Tregs, mesenchymal stem cells; immune suppression	↑ Metastasis; ↓ OS	[20]
CXCL12 (SDF-1)	CXCR4	Bone-tropic, Luminal A/B	Directs metastasis to bone/lung/liver; niche formation	↑ Bone metastasis; ↓ OS	[25,26]
